# Living in Sin? How Gay Catholics Manage Their Conflicting Sexual and Religious Identities

**DOI:** 10.1007/s10508-016-0752-0

**Published:** 2016-05-24

**Authors:** Igor J. Pietkiewicz, Monika Kołodziejczyk-Skrzypek

**Affiliations:** Faculty in Katowice, SWPS University of Social Sciences and Humanities, Techników 9, 43-126 Katowice, Poland; Kraków, Poland

**Keywords:** Gay, Catholic, Spirituality, Religion, Sexual orientation, Identity

## Abstract

Religious principles and values provide meaning and affect personal identity. They may also conflict with intimate needs and desires. This article examines how gay Catholics manage conflicting areas between their sexual and religious selves. Eight Polish gays with a Catholic background, who identified themselves as strong believers, shared their experiences during semi-structured interviews that were subjected to interpretative phenomenological analysis. Results showed that internalization of the principles taught by the Roman Catholic Church triggered a conflict when participants became aware of their homosexuality. They used a number of strategies to reconcile conflicting identities, including limiting their religious involvement, questioning interpretation of the doctrine, undermining priests’ authority, trying to reject homosexual attraction, putting trust in God’s plan, using professional help, and seeking acceptance from clergy. This study alerts mental health professionals to specific risk factors associated with experiencing a religious conflict, and offers guidelines for counseling and further research.

## Introduction

Sexual identity often exists in a dynamic interaction with other self-images such as cultural background (ethnic and/or religious), which requires that individuals manage multiple identities (Stevens, 2004). The belief systems of religiously involved people often shape practitioners’ mental and emotional states, and inform and influence general values, social axioms, and practices, including those related to health and sexuality (Pietkiewicz, 2008). While religion can be a valuable source of coping strategies by providing meaning, sense of control, comfort or support, it can also create challenges and lead to distress when its principles conflict with other aspects of life or identity (Pargament, Koenig, & Perez, 2000). This article will explore how religious socialization affected the formation of gay identity of Polish gay Catholics.

Literature shows that attitudes toward homosexuality are complex and may vary even within the same religious tradition. While some religions show little concern about the sexual life of lay practitioners or remain positive about homosexuality, most mainstream traditions are openly against homosexual acts (Helminiak, 2008). Some studies indicate that negative religious beliefs about homosexuality, shared by the community, can arouse prejudice against sexual minorities and contribute to experiencing the religious group as oppressive. For instance, Barton (2010) describes religion-driven challenges faced by sexual minorities brought up in fundamentalist Christian communities, where homosexual acts are perceived as a sin against God and condemned. Other studies also highlight the distress associated with fear of rejection for crossing the taboo by religiously oriented gays and lesbians (Mahaffy, 1996; Rodriguez & Ouellette, 2000; Thumma, 1991). Some of the potentially traumatic experiences reported by gays, including evident bullying (e.g., being teased, mocked, threatened, publicly humiliated, and ostracized) or witnessing gender-specific abuse by their peers or adults (Friedman & Downey, 1999), can be legitimized by the belief system they live in. Engaging in homosexual relationships can be even more problematic when it leads to open persecution and penalty. In some Muslim countries, for example, homosexuality is seen as immoral, sinful or an illness, strictly forbidden, and punishable by imprisonment and flogging (Jaspal & Cinnirella, 2012; Taylor & Snowdon, 2014). Yet, merely changing environment does not automatically solve the problem if gays have managed to endorse and identify with religious beliefs stigmatizing homosexuality.

Acquisition of religious values and beliefs conflicting with sexual identity involves identification with significant others who represent these beliefs. This can also take place in seemingly liberal, multicultural contexts if gays are raised in families where homosexuality is rejected based on religious principles. According to Howell (2005), recurring events involving the threat and/or fear of abandonment or rejection by significant others, especially at a young age, can have devastating results. When these relationships contain both positive and oppressive elements, this can create high ambivalence, and certain unconscious mechanisms can be used to keep positive and negative experiences apart. Because of attachment to a significant other, the child mimics his or her behavior and, later, attacks his own parts of self that had previously been directly or indirectly attacked by the oppressor. In this way, sexual minorities may develop internalized homophobia, a negative self-evaluation for being gay or lesbian (Friedman & Downey, 1999). It was long ago observed that the interaction between the individual and the environment (e.g., facing heterosexism, homophobia, oppression, and discrimination) can significantly affect the process of sexual identity formation (Cass, 1979). This, on the other hand, may lead to negative religious coping, such as punishing God reappraisals or interpersonal religious discontent (Pargament et al., 2000), and produce such pathological conditions as obsessive thoughts about guilt and sin. Various authors also note that living in an environment where homosexual expression is under attack or forbidden can lead to alienation and inhibit coming-out (Coyle & Rafalin, 2001; Flowers & Buston, 2001). For this reason, sexual behavior and related psychological mechanisms should be thoroughly analyzed vis-à-vis contextual factors.

### Attempts to Reconcile Conflicting Identities

Experiencing a conflict between ethno-religious and gay identity can significantly impact health and well-being. Participants in different studies report depressive moods, self-loathing, suicidal ideations, and feelings of social exclusion (Barton, 2010; Coyle & Rafalin, 2001; Schuck & Liddle, 2001). They also fear rejection from family, clergy, and the religious community. O’Brien (2004) coined the term “double stigma” to describe the experience of gay Christians who felt additionally rejected by other gays for being openly religious, because the LGBT environment remains critical and dismissive of Christianity. Such findings should alert mental health professionals about specific risk factors experienced by sexual minorities who are affiliated with churches condemning homosexuality. Literature shows that gays and lesbians use various strategies in their attempts to resolve their inner conflict. Various authors mention prioritizing one of these identities while repressing the other. For instance, Schnoor (2006) described how some traditionally minded Jews try to reject gay desires which interfere with their Jewish lives. He also found people who became ultra-religious to “purge” themselves of homosexual inclinations and the shame associated with internalized stigma. Another study by Mark (2008) showed that some gay Orthodox Jews seek sexual conversion therapies because they feel great pressure to conform to communal norms, and experience intense guilt and betrayal if they fail to fulfill them. Attempts to suppress conflicting, erotic impulses toward same-sex were also mentioned in reference to gay Muslims (Jaspal & Cinnirella, 2010; Kugle, 2010) or Christians (Barton, 2010; Rodriguez & Ouellette, 2000). On the other hand, some gay individuals may do the opposite and reject religion, become apostates, or declare themselves atheists. Examples of that can be found in studies on lesbian Christians (Mahaffy, 1996), Muslim gay Iranians in the UK (Taylor & Snowdon, 2014), or Canadian gay Jews (Schnoor, 2006).

There is, however, little information on how people perceive the costs of repressing either spiritual or sexual parts of their identity, what psychological mechanisms they use to reduce resulting intra-psychic tension, and how this affects their well-being. Rodriguez and Ouellette (2000) noted that many gays and lesbians have strong feelings about their religious beliefs and sexual identity, and refuse to sacrifice (reject) either part of their self. They may use compartmentalization, a short-term coping strategy to reduce intra-psychic conflicts associated with multiple, incompatible identities, by de-emphasizing one of them, depending on the context. For example, when attending a religious institution, the gay identity would be de-emphasized and, when attending a gay festival, the religious one (Coyle & Rafalin, 2001, Rodriguez & Ouellette, 2000; Wood & Conley, 2014). However, Jaspal and Cinnirella (2010) questioned the suitability of this strategy for those for whom religion constitutes a whole meaning-making system, informing their life narratives and other identities. Various authors also mention revising religious beliefs and re-interpreting religious scriptures as a way of resolving the conflict. Some gays join LGBT-affirming religious groups and create their own personal or communal spirituality (Buchanan, Dzelme, Harris, & Hecker, 2001; Rodriguez & Ouellette, 2000; Schuck & Liddle, 2001; Thumma, 1991). An additional psychological strategy worth mentioning is observed among some gay Muslims living in the UK who prefer not to define themselves as “gay.” To sustain positive self-evaluation, they make a distinction between homosexual acts and gay identity and, while they perceive these actions as wrong, they claim this does not define who they are. They also use external attribution, refuting personal responsibility for their behavior by saying they were born this way, or saw the mainstream culture as a contributing factor (Jaspal & Cinnirella, 2010). It is possible that a similar strategy could be used by Christian gays, who are taught that it is the sinful act and not the sinner who should be condemned.

Gay people reconcile their sexual and religious identities on a social level in different ways. Brekhus (2003) identified three main types of strategy in which urban gays in America manage their homosexual identity: lifestylers (peacocks), commuters (chameleons), and integrators (centaurs). Lifestylers build their self-image primarily upon being gay and they prefer to socialize exclusively in LGBT circles. Commuters lead a conventional life in the suburbs, from which they escape to satisfy their social and sexual needs with other gay people. Integrators, on the other hand, treat homosexuality as part of themselves. Their sexual identity diffuses other identities, e.g., professional.

The kind of lifestyle gays adopt, and whether or how they come-out, significantly depends on local culture (Barret & Barzan, 1996; Stevens, 2004). Flowers and Buston (2001) note that, in sociocultural environments of compulsory heterosexuality, it may be especially difficult for gay people to disclose their sexual orientation. For instance, gay Jews in the study of Coyle and Rafalin (2001), preferred not to disclose their sexual identity within their Jewish community and, like commuters discussed by Brekhus (2003), often avoided discussing relationships or sexuality-related issues, and kept a very low profile. Some complied with community expectations relating to heterosexual dating, or simply lied. In some countries, such as the Islamic republic of Iran, there is a significant prejudice and discrimination against sexual minorities, and homosexual acts are prosecuted. For this reason, contextual factors should be thoroughly examined to understand how sexual minorities integrate various parts of self, how they express these parts, and what problems they may encounter. Contemporary studies which analyze these problems are limited in scope and number. Those that exist have been primarily conducted on the American continent or in Western Europe, where there is high cultural diversity and sexual minorities enjoy more rights. No empirical studies have been found regarding Eastern Europe or the Middle East, where some countries are still significantly influenced by religion, and it would be beneficial to broaden our knowledge about experiences of LGBT people living in these areas.

### Religion and Homosexuality in Poland

Poland is an interesting showcase for the study of attitudes toward homosexuality with reference to religion. There are no laws in the country against homosexual activity; the state does not recognize same-sex unions, marriages, or allow adoption by same-sex couples. Values and norms in Polish society are strongly affected by the Roman Catholic Church. According to the Central Statistical Office (GUS, 2013), 33,384,936 people were baptized into it. Sociological analysis by Boguszewski (2015) indicates that, despite growing secularization and privatization of religious faith, the majority of the population still declares themselves to be believers (92 %) and in various degrees engage in religious practices. Forty-four percent of young respondents (aged 18–24) still report participating in holy masses, religious services, and other events organized by the Church, at least once a week (Boguszewski, 2015). In recent years, however, there has been a visible and steady decline in religious involvement of youth.

Catholic principles seem to affect the way people perceive homosexuality and think about LGBT involvement within the religious community. Roguska (2015), in her sociological report, shows that Polish Catholics do not want the church to change its attitude toward sexual minorities. Seventy-five percent are against allowing sexual minorities to use the sacrament of marriage and 52 % oppose letting them participate in Holy Communion. Despite that data show that there is a growing acceptance among Polish Catholics to living out of wedlock, pre-marital sex, homosexuality in general, divorces, and using contraception. An Internet search of local websites identified a few Polish forums and groups supporting the Catholic LGBT community, such as Faith and Rainbow (www.wiara-tecza.pl). However, no studies exist on how sexual minorities in Poland who identify themselves as Catholics cope with anti-gay sentiment in local communities or how that affects their spiritual practice. Such problems are of special interest to professionals studying human sexuality and how it affects well-being. This explorative study is an attempt to fill this gap. It aims to explore personal experiences of gay Catholics associated with their religious socialization, discovering their sexuality, and finding ways to express their sexual and religious self.

## Method

This study was conducted in Poland in 2014 and 2015. It was guided by the interpretative phenomenological analysis (IPA) framework, a particularly useful methodology for exploring people’s personal experiences and how they build their interpretations of phenomena (Willig, 2008). This methodology was selected to build a deeper understanding of how gay men, brought up in Polish Catholic families and with strong faith, developed their gay identity. Pietkiewicz and Smith (2014) explain that IPA combines ideas driven from phenomenology, hermeneutics, and idiography. It employs “double hermeneutics,” in which participants share their interpretations of phenomena under investigation, followed by researchers trying to analyze, make sense, and comment on these interpretations. Samples in IPA are small, homogeneous, purposefully selected, and data are carefully analyzed case-by-case. Willig (2008) highlights that small qualitative studies may generate hypotheses that can later be tested by methods of the hypothetical-deductive paradigm. Participants in this research described their religious socialization and its impact on how they experienced their homosexuality, experienced intra-psychic conflicts and attempts to resolve them.

The first author (IP) is non-Christian and, despite living in Poland, did not have a Catholic upbringing. His knowledge about Catholic values and norms comes from mass media and interaction with the environment where the majority is Catholic. He is also an academic teacher and certified psychodynamic psychotherapist, with substantial experience in counseling and providing psychotherapy services to gay people representing various faiths. He could thus bring a more clinical perspective into the analysis. The second author (MKS) is a psychology graduate who has been significantly involved in religious practices and the study of the Catholic doctrine, which gave us a valuable understanding of the phenomena participants referred to.

### Participants

Participants of this study were eight homosexual males aged between 24 and 45 years. Five of them had higher education, two of them secondary, and one was still a student. All were Caucasian, Polish, and brought up in families where religion played an important role in everyday life. All were significantly involved with the Church and regularly participated in religious practices at a young age and during adolescence. They all took religious lessons, attended events organized by the Church; two served as ministrants and one was involved in a Catholic youth group. All participants declared that spirituality and relationship with God was an important aspect of their lives, but five reported lesser involvement with religion in adulthood. One was in transition to the Evangelical church. Four participants currently had a relationship with another man. Only four had disclosed their sexual orientation to family. Five participants used professional help (counseling, psychotherapy or pharmacotherapy). See Table 1 for information about the interviewees, whose names have been changed to protect their confidentiality.

**Table 1:** Participants and their characteristics (*N* = 8)

No.	Name	Characteristics
1	Borys	Age 24, graduated from a middle school and is now training to be a hairdresser. Born and brought up in a large city. A few years ago, he moved to a smaller town to live with his boyfriend and his parents. His grandmother is a zealous religious practitioner, and regular Church attendance has been significant in his family; however, he stopped religious practice 8 years ago. He used counseling and disclosed his sexual orientation to family
2	Arthur	Age 25, student. Born in a small town and moved to a large city to study and live with his boyfriend. They have been in a relationship for 2 years. He was a church ministrant for a few years but has not participated in religious practices since confirmation at the age of 16 except for occasions such as family member’s baptism or marriage. He disclosed his sexual orientation to family
3	Radek	Age 25, medical doctor. Born and brought up in a large city. Has recently rented an apartment with a friend. He is single and has had one short-term relationship. His family is very religious. He was a member of a religious youth group and a church ministrant in adolescence. Although he declares himself as a man of faith, he began to withdraw from religious practices in his adolescence and now seldom goes to Church. He used psychiatric treatment for anxiety symptoms for a few years. He only disclosed his sexual orientation to selected friends but not his family
4	Konrad	Age 27, university graduate. Born in a small village and moved to a large city to pursue education and career. Single, and has never been in any relationship. He occasionally uses the Internet to arrange a sex date, after which he loathes himself. He used to be highly involved in religious practice and considered becoming a priest, but was discouraged by his spiritual guide. He has used psychotherapy. He only disclosed his sexual orientation to selected friends but not his family
5	George	Age 28, secondary education. Born and brought up in a small town. He moved to a larger city a few years ago where he works as a hairdresser. He has been in a long-term relationship with his boyfriend. For the last 6 months they have lived together with his partner’s parents. He declares himself a believer but has attended church less frequently since his adolescence. Nowadays he scarcely engages in any religious practice. He disclosed his sexual orientation to family
6	Adam	Age 32, doctoral student. Single. Born and brought up in a large city. Lives with his mother and sister. His father died when he was 15. He has never been in any long-term relationship and was reluctant to discuss his gay experiences. He was very much involved with religious practices until his confirmation. He is in transition into the Evangelical church where he attends regular meetings. His mother still attends church and his sister converted to Buddhism. He used counseling and disclosed his sexual orientation to family
7	Matthias	Age 35, university graduate. Raised and lives in a large city. In a relationship with a partner but they live separately. Previously had relationships with men and women. He and his family are deeply committed to religious practice. He used counseling and group therapy in a Catholic institution. The only family member to whom he disclosed his sexual orientation is his sister
8	Sylvester	Age 45, university graduate. Born in a small town and moved to a large city to study. He is single and shares an apartment with a friend. He has had one long-term relationship and a few short ones. He declares himself a believer but only occasionally participates in religious practices. The only family member to whom he disclosed his sexual orientation is one of his brothers

### Procedure

Following the approval of the University Committee for Research Ethics, the second author used her personal network to approach potential candidates. She personally knew two participants and others were recommended to her (chain-sampling technique). Those who met the criteria of inclusion—(a) defined themselves as gays and (b) identified a strong involvement of their families in the Catholic Church—were invited to participate in interviews. Only one person out of the ten approached did not agree to participate, without explaining why. One interview was excluded from the analysis, because it turned out during the discussion that the man’s parents withdrew from the Church when he was young and, although he regarded himself as spiritual, he never felt strongly affiliated with Catholicism. Thus, he did not meet the criteria for inclusion, which was to ensure a homogeneous sample expected of IPA studies (Pietkiewicz & Smith, 2014). Interviews were held at places chosen by the participants, usually their homes. Participants perceived the topic of the study extremely meaningful and were very willing to share their experiences.

Prior to meeting individual participants, an interview guide was created with areas to be discussed with every participant and sample open-ended questions and prompts to explore their experiences of growing up in a religious family, discovering their sexuality, and meaning attributed to homosexuality with reference to their faith. It was crucial not to make any suggestions that there might be any conflict between these areas. Instead, the intention was to encourage participants to reflect upon their experiences and share their unique interpretations, in line with IPA principles. The second author collected data using audio-recorded, semi-structured, in-depth interviews, ranging from 60 to 90 min in length. With every participant, she used open-ended questions or instructions, such as

Can you tell me about religious life in your family when you were young? How did you know religion was important for your relatives? How did your relatives and religious community refer to sexuality? When and how did you realize you were gay? How did you experience and interpret your interest in other men? What did your family and religious community think about homosexuality? How did that affect you? How do religious beliefs affect you as a gay person? How does being gay impact your spiritual/religious practice?

### Data Analysis

Verbatim transcriptions were made of all audio recordings, and analyzed using NVivo10, qualitative data-analysis software (QSR International, Burlington, MA). Consecutive analytical steps recommended for IPA were employed in the study (Pietkiewicz & Smith, 2014). For each interview, both researchers listened to the recording and carefully read the transcript several times. Individually, they produced extensive notes about the content and language use, and wrote down their interpretative comments using the “annotation” feature in NVivo10. Next, they categorized the notes into emergent themes by allocating descriptive labels (nodes). The researchers then met to compare and discuss their coding and interpretations. They analyzed connections between themes in each interview and between cases, and grouped themes according to conceptual similarities into superordinate themes and sub-themes. The aim of this study is to balance between the hermeneutics of empathy and the hermeneutics of suspicion (Smith, Flowers, & Larkin, 2009). In the Results section, the data are presented mostly at face value, highlighting similarities and differences between participants. More in-depth interpretations offered in the Discussion reflect our understanding of data, which is inevitably shaped by our professional background (i.e., clinical psychology and psychotherapy) and an assumption that unconscious processes and mechanisms exist which people may not be fully aware of (psychodynamics).

#### Credibility Checks

During each interview, clarification questions were asked to negotiate the meaning participants wanted to convey. At the end of the interview, they were also asked questions to check that their responses were thorough. The researchers also compared their interpretative notes to evaluate their understanding of the content and its meaning (the second hermeneutics).

## Results

Participants discussed their religious socialization and its impact on developing a gay identity. Significant themes repeatedly appeared in interviews and were grouped into six superordinate themes and ten sub-themes, as listed in Table 2. These are discussed and illustrated with verbatim excerpts from the interviews, in accordance with IPA principles.

**Table 2:** Constituent themes

Superordinate themes and sub-themes	No. of participants with theme
Theme 1: Growing in faith	8
Theme 2: Discovering one’s homosexuality	8
Theme 3: Experiencing a conflict	
Spiritual dilemmas	8
Fear of disappointing family	7
Anticipating rejection by the community	8
Theme 4: Seeking peace	
Reducing religious involvement	5
Questioning the interpretation of the doctrine	4
Undermining priests’ authority	6
Trying to reject homosexual attraction	3
Putting trust in God’s plan	6
Using professional help	5
Seeking acceptance from clergy	6
Theme 5: Coming-out in a Catholic family	8
Theme 6: Bearing the sin	5

### Theme 1: Growing in Faith

All participants described their religious socialization at home, school, and the Church in great detail. They characterized their families as strongly involved with religion who celebrated important religious holidays (e.g., Christmas and Easter) and treated Sundays as sacred, when the whole family would dress nicely and attend Holy Mass. Sylvester said that wearing special clothes was a way of showing respect. Borys remembered his whole family going for walks after Church and discussing themes raised during the sermon. Parents often checked how carefully children listened to the sermons. Borys also recalled saying prayers with his parents or discussing the Bible with them every evening. Seven participants recalled these childhood experiences associated with religion with positive feelings and two said they missed them.

This was fantastic. We were all together as a family. At Christmas, there would be 30 of us in a small house in the countryside. I really miss that. – Matthias (age 35)

Religious principles strongly affected the worldview and opinions shared by family members, and informed moral decisions and conduct. Five participants referred to mothers and grandmothers as guardians of the religious tradition, whereas fathers were presented as more reserved or absent. No participants talked about God with their fathers, and described relationships with them as more distant.

My mom participated in the Neocatechumenate and was very involved emotionally in faith issues. She really lived her faith. You could see she often thought about God or recalled something the priest had said, or she would say how wonderful the reading was. – Radek (age 25)

All participants were also exposed to the Catholic doctrine at schools where religion was taught. They treated clergy and religious teachers with great respect. Borys said he “perceived the catechist at the middle school as someone representing God himself because she was teaching on his behalf.” It was thus difficult to question the principles taught by them.

Exposure to intense religious socialization and resulting positive experiences led participants to ascribe special meaning to the spiritual domain. Not only were they baptized at birth, but all later attended their First Holy Communion, followed by Confirmation at the age of 16. Until then, all had regularly gone to confession. Radek and Arthur served as church ministrants and participated in religious youth organizations, trying to embody their faith in everyday life and follow Catholic principles in distinguishing what was right, moral, or sacred, and what was immoral or sinful.

### Theme 2: Discovering One’s Homosexuality

All participants said that acknowledging their attraction to same-sex was challenging and even challenging to their self-image. Borys recalled starting to “discover something in himself” but could not understand why he was different from his classmates, and searched information about homosexuality on the Internet to familiarize himself with the topic and understand his own desires.

They talked about boobs and asses all the time. I didn’t find that interesting and had to hide my feelings. I felt weird, embarrassed. They were all thinking and talking about girls and I felt different. So I started searching the Internet to understand. – Borys (age 24)

George, however, claims he always found other boys interesting and preferred to spend time with them. As a teenager he decided he ought to have a girlfriend, but found this experience unsatisfactory. Later, he visited gay clubs and dated boys with whom he established intimate relationships.

I felt weird in those places [clubs] as it was completely new to me, but week after week I began to get used to that environment. Later on I even began to date guys and have, you know, hanky panky. – George (age 28)

Other participants claimed they were unaware of their sexuality for a long time. Interestingly, four of them reported studying the Bible even more in adolescence because they were seeking answers to existential questions or to feel closer to God. Then, between the ages of 21–24, they experienced a “sudden revelation” or were confronted by someone to whom they were attracted. Matthew said a befriended priest suggested he might be gay, and Arthur recognized his gay desires when he fell in love with a man.

### Theme 3: Experiencing a Conflict

All participants experienced at different moments of their lives intense conflicts between developing sexual awareness and needs, and their religious beliefs and aspirations. Two found ways to come to terms with their sexual identity, but others experienced emotional turmoil (see “Theme 4: Seeking Peace” section). Homosexual inclinations were threatening because they were seen as sinful, disappointing to family, and put them at risk of social discrimination.

#### Spiritual Dilemmas

Participants highlighted that although the Catholic Church officially does not condemn homosexuals as people, intimate behavior with same-sex was regarded as a sin against God. Gays were expected to keep sexual abstinence, even when living in a loving relationship with a partner. Internalization of religious principles made participants feel extremely guilty and fear eternal condemnation. On the other hand, being unable to satisfy their needs for love and intimacy evoked frustration and anger toward the doctrine. Those who confessed to having an intimate life with another man often heard it was sinful and immoral. Borys described a confession that he found traumatic.

I confessed everything to the priest. When I started to say that I had a partner and we engaged in sex, the priest said I had to stop, because God did not like that. He said this sin was worse than murder. At that point I decided not to go to confession anymore. – Borys (age 24)

Sylvester, Adam, and Matthias had similar experiences and felt guilty for not getting absolution. Sylvester was also angry at one encounter which led to him avoiding discussing his personal life with priests.

I had problems with confession especially when I felt bad about something. Once I talked to a priest at the famous Luminous Mount monastery, but he really discouraged me. I left the confessional before getting absolution. The priest called me a pervert or something. He shouted at me and I didn’t go to confession until my pilgrimage to Assisi five years later. – Sylvester (age 45)

#### Fear of Disappointing Family

Participants said the teachings of the Church strongly affected their relatives’ values and expectations about social roles. Receiving a sacrament of marriage, conceiving offspring and rearing them according to Catholic norms was highly valued. Fear of disappointing loved ones was often an obstacle against coming-out (described in Theme 5).

I certainly wouldn’t meet their expectations, because my parents wanted me to have a family. They wanted grandchildren. That would destroy their dreams. – Radek (age 25)

While concerns or disappointment Radek attributed to his parents may be typical of any family irrespective of religious background, he and Arthur also discussed religious consequences. Both were convinced their mothers would wonder if they could meet in heaven. Participants claim that concerns about the afterlife are crucial for all believers.

#### Anticipating Rejection by the Community

All participants referred to social stereotypes about gay people, strengthened by media reports which, they believed, often viewed gays as “effeminate” or “camp” in the way they dress, talk or move. Our participants did not identify with that image, and cared about behaving “normally,” meaning “manly.” Borys claimed that society, including priests and religious teachers, associated gays with promiscuity, immoral behavior, sexually transmitted diseases and the inability to establish long-term relationships. He was ashamed about disclosing his sexual orientation and anticipated rejection when religious teachers, nuns, or priests made negative comments about gay life during classes or sermons.

I felt rejected during religious lessons, even though the catechist was not directing his comments to me. Obviously, no one knew I was gay. I was 13 then, and you know… faggots, dykes, etc. No one would officially admit being homo. When people made jokes about gays it really hurt. Now, when I listen to what priests say about gay people I feel like standing up and shouting that it is not true. Look at me and my boyfriend! We do not change partners like gloves, I am not HIV positive, I don’t have AIDS or hepatitis! – Borys (age 24)

Radek was also hurt by a comment he heard in the religious community that “love cannot be born in a swamp.” Adam interpreted social prejudice against gay people in terms of scapegoat mechanism. He thought people ascribed to the LGBT community all kinds of sinful qualities they would not like to find in themselves.

### Theme 4: Seeking Peace

All participants tried to resolve inner conflicts between their religious values or aspirations, and growing gay identity. They used strategies such as reducing religious involvement, questioning the interpretation of the doctrine, undermining the authority of priests, and putting trust in God’s plan.

#### Reducing Religious Involvement

Participants said they were frustrated and angry, and felt guilty and rejected by the Catholic Church, because its doctrine forbids homosexual love and relationships, treating these as sinful actions. Five participants gradually withdrew from religious practices and attending church, because they felt unwelcome. Radek talked about his disappointment and sadness:

I don’t see the problem. I don’t think it matters who I love. I am just sorry the Church can’t open its gates to all mankind, including homosexuals. We are not openly invited. We feel excluded, like sinners. – Radek (age 25)

#### Questioning the Interpretation of the Doctrine

Another way of coming to terms with one’s sexual identity included attempts to challenge the Bible hermeneutics provided by the Church in relation to homosexuality. Matthias maintained that the negative attitudes of the Church toward homosexuality were based on the Old Testament, which he saw as irrelevant in modern times. He was convinced that there were no direct references in the New Testament that would condemn homosexuality as sinful behavior.

There are so many things in the Old Testament that are impossible to apply in our Catholic faith. That was a completely different world, so it was also written in a specific way. I rely on the New Testament and, although I am not a Bible expert, I don’t think there is really anything against homosexuality there. – Matthias (age 35)

#### Undermining Priests’ Authority

Six participants expressed doubts about priests’ personal conduct, and two of them exhibited strong hostility toward clergy. Adam said he was once verbally attacked and thrown out of confession, but he admitted to teasing the confessor, despite knowing he would not agree with his views.

I was angry when I went to see him and tried to challenge his views. I told him I was in love with a married guy and that Jesus also had a special relationship with some of his students. He screamed at me and threw me out. That was foolish of me and I wouldn’t do it again. I don’t need to prove anything to them [priests]. – Matthias (age 35)

Another participant belittled priests as moral authorities. He was convinced they themselves committed sins by engaging in inappropriate sexual relationships. He claimed that one of his confessors was overtly interested in hearing details about his masturbation and intimate contacts and was convinced the priest was aroused by those stories. This disgusted him and he felt they had no right to tell him what behavior was virtuous and what was sinful. This helped him cope with anticipated critique and rejection.

Many priests are heterosexual, but shouldn’t they respect celibacy? It really bothers me to think where that priest has put his hand before touching the holy host. I stopped going to confession. Why should I confess to a stranger who may be a greater sinner than me? Because that does happen. Who is he to tell me how to be a good man? – Sylvester (age 45)

#### Trying to Reject Homosexual Attraction

Three participants felt awkward about being homosexual. They shared negative views about the gay community and sought individual and/or group therapy hoping to suppress their attraction toward other men so they could establish traditional families. Instead, they found themselves confronted with conflicting needs and desires. Matthias attended group counseling for gay men held by clergy. He said it was a valuable experience because he learned to express emotions and feelings, gained more insight into his behavior, and fell in love with another group member. He thought participation in the group helped him embrace his homosexuality, even though he initially hoped to change it. Adam also came to terms with his homosexuality, but Konrad never managed to accept himself. He often experienced guilt, shame, and self-loathing. He was unable to suppress his sexual drive and engaged in occasional sex and compulsive masturbation after which he became auto-aggressive and sometimes had suicidal thoughts. He also admitted to being homophobic, and strongly attacked in himself a desire for emotional involvement with other men.

There are days when I feel depressed and isolated at home. I don’t wanna see any friends; I have a beer or two and watch these [pornographic] pages for hours. Sometimes I enter a chat room to hook up for a sex date. I help myself [masturbate] to reduce tension but later I feel really bad, dirty. I hate myself for all that. When I have a date it is just for sex, and I never meet the guy twice. I prefer not to know their names or what they do. I cannot imagine getting emotionally involved with another man, not to mention establishing a relationship. That is a sin. – Konrad (age 27)

#### Putting Trust in God’s Plan

Trying to accept one’s homosexuality often required rejecting the doctrine presented by the Catholic Church, according to which two men engaging in sexual acts would be eternally condemned. However, all participants stressed that their individual relationship with God was still important. Three questioned whether their love for another man was a sin and all were searching for meaning, why they were born homosexual. Adam speculated that if “God created mankind in his own image,” then being attracted to another man must also have a divine aspect and should be acceptable. Radek imagined God as benevolent and hoped any of his faults would be forgiven, as long as he followed his conscience.

If I am a good man and live according to my conscience, then God will forgive me everything. Such an omniscient and powerful person cannot be unable to forgive. – Radek (age 25)

Interestingly, he refers to God as a person endowed with certain qualities; perhaps, the ability to forgive is something he would hope to receive from his parents if only he were able to disclose his sexual orientation to them.

#### Using Professional Help

While four participants reported using some sort of counseling or psychotherapy because they experienced a conflict associated with their homosexuality and/or had problems with coming-out, only Borys said he expected support rather than trying to change his sexual behavior. Unlike his mother, he did not believe he could be “cured from that affliction.”

It was the first time I felt that someone really cared about me and was interested in my feelings. I felt rejected by my mother because she couldn’t accept I was gay, and the therapist really listened to help me overcome my fears and accept myself. I didn’t want treatment to “cure” my homosexuality. – Borys (age 24)

Radek used psychiatric medication for 6 years to treat his panic attacks but was unable to understand reasons for his anxiety. He said he was only worried about how his family would react to learning that he was gay.

#### Seeking Acceptance from Clergy

Six participants said they hoped for support from priests but were discouraged by their expectation of hostile attitudes toward gay people. While five participants had negative experiences during religious lessons or confession (see “Theme 3: Experiencing a Conflict” section), three of them reported positive responses. Sylvester described the relief he experienced from the supportive words of his confessor:

I was complaining about everything and told him all these sins, etc. He just looked at me and said: “You know, St. Francis was not much better than you at this age.” This was all he said to me and he granted me absolution. That was such a relief. – Sylvester (age 45)

Adam hoped that Catholic priests could change their attitudes toward homosexuality and he often provoked tense discussions with them. Years later, he established a friendly relationship with a pastor of an Evangelical church whom he found more accepting and open-minded, which encouraged him to convert. Konrad also perceived his spiritual director as uncritical and understanding, which only revealed the discrepancy between his own harsh super-ego and self-hatred for being gay. Konrad admitted that gave him some sense of control—no one could hurt him more, because he was his own most critical judge.

### Theme 5: Coming-out in a Catholic Family

Four participants reported disclosing the fact of being gay to their families. One kept it entirely secret and three only revealed it to a selected sibling. Reluctance to coming-out was associated with anticipated reactions. All participants were convinced that it was difficult for their parents to accept that they were gay. Borys said it was a shock for his mother when he came-out during a quarrel. Feeling rejected by her made him seek support in counseling.

When I was 16 we had a fight about my school results and behavior. She said something like: “Perhaps you are a faggot,” and I said, “You bet I am.” It was a shock for her. When she realized what this meant she said she didn’t want to know me, that she didn’t have a son anymore. She ignored me and didn’t speak to me for a month. It was the most difficult time in my life. – Borys (age 24)

Adam thought his mother still believed he would change and establish a “normal” family. He attributed her inability to accept his homosexuality to being “programmed by the Church.”

She referred to the assumptions made by the Church that this is a terrible sin, that these people will never be happy, risk HIV, AIDS, and never establish permanent relationships. – Adam (age 32)

George believed his parents would rather think of him as a bachelor who was preoccupied with work and uninterested in relationships, rather than a homosexual living with a partner. He thought that was unfair and resulted from the shame of having a gay son.

My parents prefer me to stay single, saying: “You would be better alone.” Then I ask them: “But why? Shall I sit with you on a couch, drink coffee and watch TV or go to Church for the rest of my life? Why should I be alone if I can be happy with someone?” There is someone I love, who supports me. My mom would find it easier to accept if I stayed a bachelor than to think of me as a homosexual living with another man. – George (age 28)

### Theme 6: Bearing the Sin

All participants believed the Church was unequivocal toward same-sex relationships, seeing them as sinful. Those who accepted homosexuality as part of who they were and realized their needs for intimacy with a partner, claimed they did not regard gay identity as a sin. To achieve that, they rejected conventional interpretations of the doctrine and adopted alternative hermeneutics. They also perceived that they had to put trust in God’s benevolence. On the other hand, Borys questioned whether such conscious attempts could resolve deep-rooted conflicts.

I wondered about converting to another church, but would that help? Even if I changed faith, I have been raised according to this doctrine—that only heterosexual relationships are acceptable—since childhood. This is what I was taught by priests and religious teachers. That concept permeates my being. – Borys (age 24)

Those who limited their participation with the Church said that confession made them confront the anti-gay attitudes they tried to reject. Four participants perceived that homosexual relationships were inappropriate. Konrad was full of shame and guilt for committing the same sin repeatedly (i.e., engaging in homosexual activities). George tried to compensate God for his “sinful life” by performing good acts (e.g., giving financial support to his sister and helping her raise her children).

God does not like me to go with men. I go to confession, but I know I will repeat my sin sooner or later. I try to make it up to God by good deeds. I help my sister by giving her money. I tell myself that, as I am gay, I can support her financially as I don’t have my own kids. – George (age 28)

Borys also expressed his great wish and sadness for not being able to receive a blessing for his relationship. He thought it would show society that such relationships can be full of care, love, and respect.

## Discussion

This study explores how Polish gay Catholics establish their gay identity with reference to the religious tradition in which they were brought up and the beliefs they endorsed and internalized during socialization. The context of this study is unique, because Polish society is generally homogeneous, meaning that the majority shares the same race, language, societal norms, and traditions, and declare themselves to be Catholics. Despite globalization, Poles have relatively low exposure to cultural differences in terms of ethnic or religious backgrounds compared to many countries in Western Europe or America. Our participants were raised in Catholic families where regular religious practice and living the principles of faith were emphasized. Religion affected their values and social axioms (including eschatological beliefs), and was perceived to be a significant channel through which people not only expressed their spiritual needs, but also the need for affiliation and closeness. Participants in this study provided interesting examples about how their family life was organized around religious practice (e.g., parents read and discussed biblical stories with their children; they attended services together and celebrated religious holidays). However, the very faith that often helped find meaning, spiritual strength and solace, at some point triggered a conflict when participants began to experience same-sex attraction and realized they might be gay. Sexual minorities are probably not alone in experiencing conflicts associated with faith. Heteronormative Catholics may also struggle with moral dilemmas about pre-marital sex, committing adultery, divorcing, using in vitro fertilization, etc. What is, perhaps, special about being gay is that the conflict refers to identity, and not merely behavior (despite the fact that some gays try to separate homosexual acts from being gay, e.g., gay Muslims in the UK studied by Jaspal and Cinnirella [2010]).

Discovering attractions toward same-sex in an environment characterized by anti-gay sentiment evoked anxiety. Similar experiences were reported by homosexual Jews (Coyle & Rafalin, 2001; Mark, 2008; Schnoor, 2006), Muslims (Kugle, 2010), or Christians studied by Barton (2010), Mahaffy (1996), Thumma (1991), or Rodriguez and Ouellette (2000). Our participants also shared similar concerns to gay Asians (Chan, 1989; Wang, Bih, & Brennan, 2009) or Jews (Mark, 2008), who worried about disappointing or being rejected by families for failing to fulfill social expectations to establish a “normal” family, and procreate. Feeling “different” and confronted with forbidden desires required participants to seek ways to resolve inner conflict. Literature identifies strategies used by gay people, such as trying to reject their gay identity or religious identity, using compartmentalization (an unconscious psychological defense mechanism used to avoid cognitive dissonance by keeping two identities separate), or integrating conflicting identities (Rodriguez & Ouellette, 2000). However, as many of these studies are sociological, there is little information about the mental processes and psychological mechanisms involved in the strategies investigated. For instance, if gays reduce their involvement with religion (even to a minimum) to find intimate fulfillment with a partner, how do they cope with internalized beliefs conflicting with their sexual behavior? Does rejecting these beliefs on a conscious and rational level free them from experiencing emotions previously triggered by the described conflict? What psychological processes are required to integrate conflicting identities?

### Controlling Desire

Various authors report that gays and lesbians may attempt to suppress homosexual needs (Barton, 2010; Rodriguez & Ouellette, 2000; Schuck & Liddle, 2001). Our participants were no different. They tried to reject their unaccepted desires, with little success. Unable to “sustain purity,” many of them felt guilty and sinful, apparently due to internalized values and beliefs about sexuality shared in their close environment. Participants ascribed their significant others with traditional religious beliefs concerning intimate life (its purpose and normative expressions of sexuality) and could not differentiate between opinions about sexuality presented by their parents or grandparents from those of the Church. Self-hate in this case could result from an identification with the oppressor (Friedman & Downey, 1999) mentioned earlier. It is not clear which factors were more stressful for our participants—the reluctance toward gays attributed to the Church and its leaders, or the attitudes toward sex and spirituality ascribed to significant others. All our participants described their families as highly involved with religion. There was only one person whom Brekhus (2003) would call a lifestyler, but he was excluded from the analysis because it turned out his family had a relaxed attitude toward religious practice and withdrew from the Church when he was still a child. Positive identification with supportive parents might have helped him cope better with an oppressive culture. This requires further analysis by comparing religious individuals from more fundamentalist and liberal families.

### Reducing Religious Involvement

Another coping strategy our participants used was to limit their religious involvement, e.g., Church attendance and confession (Barton, 2010; Coyle & Rafalin, 2001; Rodriguez & Ouellette, 2000). However, they also admitted to a sense of loss—they really missed participating in community events and sacraments. There is no evidence that rejecting organized religion can solve religious conflict if people still carry their internalized beliefs and norms. For example, one participant (Borys) said that although he could leave the Church and consciously reject conflicting beliefs, they were still with him, because they had been imprinted in his mind, since childhood. It is possible that those who leave still feel guilty about breaking a taboo (whether consciously or not) and try to make it up to God, the world, or other people. There may be additional unconscious motives for leaving, besides the conscious ones (i.e., refraining from a community in which one feels unwelcome). Some gays may project their own (previously internalized) negative feelings about sexual minorities onto priests and, by avoiding contact with clergy, gain control over their own ambivalence. While projection can help people deal with threatening parts in themselves, it can also distort reality (i.e., not all Roman Catholic priests are hostile toward homosexuality) and influence relationships with the external world. For instance, many of our participants made no further attempts to seek spiritual guidance from priests, even though some might have been potentially more supportive.

Participants also reported conflicting relationships with significant others—a few referring to distant, uninvolved, critical fathers or rejecting mothers. Transferential feelings and emotions may influence how sexual minorities perceive the Church, its ministers, and God. Transference, the mechanisms of projection and projective identification likely to be involved here, are thoroughly discussed in the psychoanalytic literature (Gabbard, 2014). This interpersonal dynamic can fuel already existing tension associated with an oppressive and victimizing environment.

### Undermining Religious Leaders’ Authority

Our study identified a unique coping mechanism, not mentioned in other studies, which involves undermining the moral authority of the church and clergy. This is an example of negative religious coping (Pargament et al., 2000), expressed by interpersonal religious discontent. By using devaluation, some participants felt justified in questioning priests’ eligibility to make comments about gays’ moral conduct. This may reflect the oppressor-victim dynamics mentioned earlier, in which various conflicted parts are likely to activate at different moments (Howell, 2005). Participants sometimes felt oppressed by priests’ anti-gay attitudes (e.g., making harsh comments in public or refusing to grant absolution) and at others they could themselves become aggressors.

### Developing Autonomy

Reconciliation of conflicting identities may only be possible via differentiation and separation. In this study, and others (Jaspal & Cinnirella, 2010; Rodriguez & Ouellette, 2000; Schnoor, 2006; Schuck & Liddle, 2001; Thumma, 1991), gays report actively exploring their own spirituality and trying to de-legitimize official interpretations of the doctrine, seeking alternative hermeneutics. In other words, they symbolically separate from authority, just as adolescents need to rebel from their parents, questioning their values or ways of life, in order to build autonomy. They compare and contrast their own with other families, and make personal, mature choices in life. This natural process enables adults to create an integrated, multi-dimensional image of their parents which helps to identify with them in some areas and disagree in others, understanding their parents’ strengths and weaknesses, and perceiving them as separate and unique individuals. What also helps people differentiate and separate is establishing new relationships of dependence (with another person, spiritual guide or group). Identification with and dependence on new objects helps individuals revise old relationships and beliefs. A similar individuation process may occur in people who join alternative religious groups affirming gay spirituality. Various authors (Barret & Barzan, 1996; Rodriguez & Ouellette, 2000; Thumma, 1991) highlight the significance of establishing new affiliations with such communities. According to Barret and Barzan (1996), gay people can feel liberated from the Church as an external authority. In our sample, only one participant was in transition to another Church, while others simply reduced their religious involvement. Although they felt physically separate from their religious community, they still endorsed its beliefs and expressed doubts about religious consequences of the lifestyle they had chosen.

### Using Benevolent Reappraisal

Our study identifies expressions of positive religious coping strategies (Pargament et al., 2000) used by participants to reconcile their gay and religious identities. These involve benevolent religious reappraisal to make meaning of being homosexual, seeking comfort and reassurance through God’s love and care, or using spiritual counseling. Similar strategies were also reported by gay Muslims who believed Allah was merciful and created them as they were (Jaspal & Cinnirella, 2010). Many of our participants ascribed God with human qualities, e.g., loving, caring, just, and also judging and forgiving. Rizzuto (1979) explained that the personal image of God is often in a dynamic relationship with significant others. In an oppressive religious environment which attacks parts of identity (homosexuality), persecutory, punitive elements in this image of God may be found. Howell (2005) says, however, that people tend to maintain and protect the situation of tenderness by dissociating memories of the abusive situation (i.e., protecting the good from being overwhelmed by the bad). Similarly, the image of God can also be split into a benevolent, idealized object, and an oppressive, threatening one. Putting trust in God as an idealized object may involve projecting the split oppressive part into the Church and its representatives. This could describe examples in this study. Additionally, some participants described sometimes feeling they were in contact with the punitive aspect and used punishing God reappraisal. Unfortunately, this study was limited by research questions, and participants’ coping strategies were not explored in relation to their personal image of God or attachment styles. Exploring these areas in sexual minorities living in various cultural contexts would be an interesting area for further investigation.

Although literature lists these main strategies as alternatives, this study shows that some participants used a variety of these strategies simultaneously or sequentially, if one was found ineffective. Certain strategies could also fade and become reactivated (e.g., Konrad limited his religious involvement but had bouts of frequent Church attendance to cope with his guilt and self-loathing, after which he would immerse himself in work again and stop religious practices). Further analysis could identify the circumstances in which gays activate these strategies.

Sexual minorities were found in this study to experience intense internal conflicts relating to their religious beliefs and affiliation. Because of potential risks associated with ego-dystonic^1^ homosexuality, screening tests for sexual behavior and psycho-education should be fostered in religious communities. Those who exhibit conflicts relating to homosexual attraction should be encouraged to use counseling. Using spiritual guidance offered by priests who express positive attitudes toward gays and lesbians should also be encouraged. Clergy should be educated about the potentially devastating effects of openly expressing prejudice against the LGBT community by people who represent authority. Therapists, on the other hand, are encouraged to explore spiritual conflicts with their clients, and analyze meanings ascribed to being gay, with reference to religion. They should also encourage clients to explore their spirituality by examining their religious beliefs from different reference points (e.g., cultural, political), and seek religious support groups for sexual minorities. Psychologists should examine clients’ transference on the religious institution and clergy, individual image of God, and coping strategies in relation to their clients’ personality and relationships with significant others.

This IPA study, by its nature, analyzed a small sample idiographically. Although no claims can be made about the general applicability of our findings because representation is not an issue in qualitative studies, certain areas are highlighted that can be of interest to community health psychologists, counselors, psychotherapists, and researchers who take on further psychological research into LGBT spirituality and sexual behavior. This study sample consisted of individuals with deeply religious upbringing, but it transpired that most of them limited their religious involvement when they found it conflicting with their gay identity. Further comparative studies should be carried out with people who remain active practitioners, convert, or join alternative religious groups specifically oriented toward the LGBT population. This sample is also disproportionately made up of highly educated men. How they cope with conflicting identities may vary from those with less education, lower social capital, or levels of social agency. Such differences can be analyzed through comparative studies. The sample was also limited to individuals who lived in large cities. It is likely that gay inhabitants of rural areas (known for higher religious involvement and more collective culture) experience problems discussed herein with greater intensity. Bell and Valentine (1995) explained that it is especially difficult to disclose sexual orientation in such environments. The interviewer in this research was not gender matched to the participants and they had not been informed about her sexual orientation. It is difficult to speculate how this affected their disclosure. Taking into account how they spoke about male authority (their fathers or priests) it is possible that having a female interviewer, of a similar age to most participants, helped some of them open up and disclose their attitudes explicitly. Finally, strategies to cope with conflicting sexual and religious identities, and consequences of using them, should be analyzed quantitatively. They could also be explored in reference to personal image of God or numerous psychological variables such as personality traits, attachment style, or self-compassion.

There is little psychological research on how gay Catholics brought up and living in highly religious environments establish their sexual identity. Most studies relating to conflict between ethno-religious and sexual identities are dated, sociologically oriented, or involve other denominations and more multicultural countries. This study may be the first Eastern European empirical research exploring intra-psychic conflicts associated with establishing a gay identity vis-à-vis Catholic identity, and discussing strategies used by participants in their attempts to reconcile conflicting identities.

## Footnotes

1 Ego-dystonic denotes aspects of one's thoughts, impulses, attitudes, and behavior that are experienced as repugnant, distressing, unacceptable, or inconsistent with the rest of the personality.
